# Comparison of Different Microalgae Biomass Typologies Used in Rotifers Enrichment for Zebrafish (*Danio rerio*) Larvae Nutrition

**DOI:** 10.1155/anu/5351038

**Published:** 2025-06-06

**Authors:** Daniela T. de Castro, Matthew Castaldi, Gil Martins, Tamára Santos, Hugo Pereira, Patrícia Diogo, João Varela, Paulo J. Gavaia

**Affiliations:** ^1^Centre of Marine Sciences, University of Algarve, Faro, Portugal; ^2^GreenCoLab - Associação Oceano Verde, University of Algarve, Faro, Portugal; ^3^Faculty of Sciences and Technology, University of Algarve, Faro, Portugal; ^4^Faculty of Medicine and Biomedical Sciences, University of Algarve, Faro, Portugal; ^5^Necton S.A., Olhão, Portugal

## Abstract

The use of enriched rotifers with industrially produced microalgae represents a valuable tool for the enhancement of zebrafish larval nutrition and increased biological performance. Currently, a monoculture of microalgal species (*Nannochloropsis* sp.) in form of liquid paste is routinely used for rotifers enrichment for zebrafish larvae feeding; however, the most adequate typology (i.e., paste or freeze-dried) of the industrially produced microalgal biomass is still controversial. This work aimed to compare the effects of rotifers enriched with three different industrially produced microalgae species (i.e., *Nannochloropsis oceanica*, *Tetraselmis chui*, and *Tisochrysis lutea*) using paste and freeze-dried powder. Enriched rotifers were provided as feed during larval growth and the impact on growth and survival was evaluated. The use of enriched rotifers with both paste or freeze-dried microalgae improved growth compared to larvae fed exclusively with commercial microdiet. Larvae fed rotifers enriched with *N. oceanica* and *T. chui* attained higher weight and length both at 15 and 30 days postfertilization (dpf), while the use of microalgae in paste contributed to greater larvae lengths when compared to freeze-dried. The experimental results in this study revealed that *N. oceanica* and *T. chui* in paste are the most suitable microalgae forms to be used in zebrafish larvae nutrition and in the improvement of enrichment methodologies for rotifers.

## 1. Introduction

Zebrafish, *Danio rerio* (Hamilton, 1882), is the second most used animal model due to its many advantages such as a fully sequenced genome, fast larval development, and short generation time [1]. Moreover, zebrafish present several technical advantages such as well-established target mutagenesis technologies, embryo transparency, rapid growth, and high fecundity [2–4]. Because of those multiple advantages, the use of zebrafish as a model species is increasing worldwide. Due to the importance of nutrition and its impact on the number of laid eggs, growth performance, and welfare [5], many studies are now focusing on the understanding of zebrafish nutritional requirements and the optimization and standardization of dietary protocols [6–8]. The lack of an adequate nutritional profile and standardized protocols leads to high experimental variability intra- and interfacilities. Thus, a better understanding of zebrafish nutritional requirements along with the adequate choice of the best types of food (microdiet, live feed, or a combination of both) and methodology of application for each life stage, is a highly relevant topic. The use of live feed, such as *Artemia* nauplii and rotifers (*Brachionus plicatilis*) enriched with microalgae, applied to the early life stages of zebrafish was adapted from marine aquaculture practices. Although zebrafish is a freshwater species, it has altricial larvae, like most marine fish larvae, with immature organs and the use of rotifers enriched with microalgae proved to improve survival and promote a rapid development [6]. This performance is thought to be related to predigestion of nutrients by enzymatic processes in the rotifers [9].

Microalgae are valuable nutritional resources, presenting a balanced profile regarding proteins, lipids, and carbohydrates as well as relevant quantities of carotenoids and vitamins, important for (zebra)fish larvae nutrition [10]. Different microalgae species have distinct nutritional profiles and can be strategically selected as functional food according to their characteristics. Microalgae production, however, presents challenges due to its high cost, trained personnel, time consumption, seasonality, and the need for specific conditions [9, 11]. In aquaculture, microalgae can serve as a nutritional tool either directly or indirectly. Indirectly, they are employed in live form through the green water technique, where they help maintain the nutritional quality of live prey [12] and promote changes in the visual contrast of the medium [13]. Additionally, they contribute to the diversification of the microflora in both the tank and the larval gut. However, the use of live microalgae requires careful management of nutrients and water quality, thus, limiting the time window for their usage.

An alternative is the use of processed microalgae, which are easier to handle and store [14]. Direct applications include incorporation into microdiets or enrichment of live prey. Powdered or freeze-dried microalgae generally have lower levels of sensitive nutrients, such as essential fatty acids, pigments, and certain vitamins. These components are prone to degradation during the drying process and their bioavailability may decrease further upon rehydration, compared to fresh or paste forms of microalgae [15]. In contrast, microalgae in paste form undergo less processing, allowing them to retain nutritional profile closer to the unprocessed microalgae. However, freezing this form can cause cell wall damage, leading to nutrient leakage and faster organic breakdown [16]. Microalgal concentrates are particularly favored due to their ease of use, cost-effectiveness, and positive outcomes [9, 11]. Furthermore, pastes and powders are commonly used to modify the nutritional profiles of rotifers [9], despite the fact that the use of freeze-dried microalgae form requires more time to prepare the enrichment medium than the use of paste forms.

In zebrafish, *Nannochloropsis* sp. is commonly provided during early larval stages [17, 18] as live prey enrichment, since it has a balanced biochemical profile and high polyunsaturated fatty acid (PUFA) content, particularly eicosapentaenoic acid (EPA, 20:5*n*−3), which is essential for both larvae development and for rotifers culture [19]. However, to the best of our knowledge, only spirulina (*Arthrospira platensis*) was investigated in the scope of zebrafish nutrition, being used for enrichment of rotifers with encouraging results [20]. In the past decade, major biotechnological advances have originated the availability of a wide range of microalgal species with different and valuable properties (e.g., antioxidants, immunostimulants, probiotics, anti-inflammatory, etc.) that can be used to optimize zebrafish nutritional protocols.

Rotifers are zooplanktonic invertebrates used as live feed for fish larvae due to their adequate size for larval mouth gap and possess slow locomotion to stimulate predation, which is known to reduce stress related to captivity and improve animal welfare [21–24]. The use of enriched live feed is relevant not only for proper nutrition but also as environmental enrichment. Additionally, rotifers are easy to grow and are generalist filter feeders, allowing nutritional enrichment with target nutrients relevant for larval nutrition [9, 25–27]. The use of microalgae as nutritional enrichment can also be applied to *Artemia* nauplii (i.e., GF strain, 450–600 μm), but is not applicable to *Artemia* instar I nauplii, since at this stage the mouth is still not open [28]. Despite nauplii having an adequate size for larval mouth gap, it contains a poor nutritional quality and is not suggested to be used in zebrafish larvae, since it promotes an increase in the incidence of skeletal malformations [29] and promotes size dispersion among larvae [30]. *Artemia* nauplii only can be enriched at the instar II stage, at which point, they are too large for the early fish larvae mouth size.

Rotifers became the most useful live feed for zebrafish larvae ingestion (120–300 μm) due to their slow swimming speed and tolerance to high culture densities. Rotifers are commonly fed with a monoculture of microalgae [31], but the use of other nutritionally valuable microalgae species to enrich rotifers can be beneficial during early larvae development. In this sense, relatively high contents of essential amino acids such as leucine (11%) and valine can be found in *Dunaliella bardawil* and *Arthrospira platensis* [26, 32], respectively. These essential amino acids cannot be endogenously produced and are necessarily obtained through the diet, having a critical biological importance for optimal growth [33]. Also, *Tetraselmis* sp. is rich in high-quality proteins that benefit larvae development, while *Tisochrysis lutea* has anti-inflammatory activity along with high docosahexaenoic acid (DHA, 22:6*n*−3) content, which is essential in fish nutrition and particularly during early life stages, since the development of their sensory organs depend on essential fatty acids [34].

Industrially produced microalgae are available in different typologies of biomass, such as powders (freeze-dried or spray-dried), pastes, and liquid concentrates. The selection of the most adequate microalgae biomass typology to be used in the enrichment of rotifers for zebrafish nutrition and is essential to improve its application and standardize dietary routines.

The objective of this study was to evaluate zebrafish larval performance when fed with rotifers enriched with different industrially produced species of microalgae (i.e., *Nannochloropsis oceanica*, *Tetraselmis chui*, and *Tisochrysis lutea*). Each microalgae species was also tested in two biomass typologies (paste or freeze-dried powder) to evaluate what is the most adequate for zebrafish larvae growth and survival.

## 2. Materials and Methods

### 2.1. Culture of Rotifers

**Figure 1:**
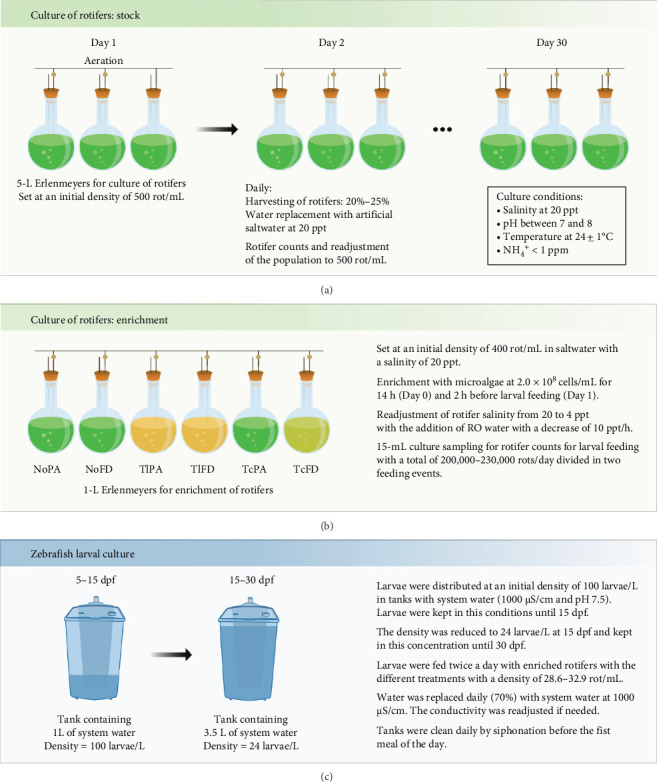
Methodology scheme (A) culture of rotifers, (B) enrichment, and (C) zebrafish larval culture.

Rotifers (*Brachionus plicatilis*, *type L*) were grown in a semicontinuous culture using 5-L Erlenmeyer flasks at 20 ppt, with a temperature controlled at 24 ± 1°C under slight aeration conditions as described [35]. Artificial saltwater at 20 ppt was prepared daily, using tropic marin synthetic salt (TMC, Portugal). To maintain the culture quality, 20% of total water volume was renewed daily and replaced with artificial saltwater. Additionally, 20% of the rotifer culture was harvested daily for larval feeding purposes. The rotifers were washed through a 300 µm sieve to remove debris and microalgae agglomerates and residues. The culture density (rotifers/mL) was determined daily, by collecting a sample of 15 mL, to maintain the culture density at ~500 rotifers/mL. Rotifer counts were performed under a stereomicroscope Leica MZ6 (Leica, Germany). Rotifers were fed (thrice a day) with Phytobloom Green Formula (Necton S.A., Portugal), a concentrated product of *Nannochloropsis oceanica* using a final volume of 1.6 mL/10^6^ rotifers (Figure 1A).

### 2.2. Enrichment

To achieve a final concentration of 18% in terms of cell density, 0.4 g of microalgae powder or frozen paste, sourced from Necton S.A (Olhão, Portugal), was added to saltwater. The mixture was homogenized using a vortex mixer, ensuring uniform distribution of the microalgae. The enriched solution was then stored at 3°C in a refrigerator. Separately, rotifers were harvested and concentrated using a 55 µm sieve to reach an initial density of 400 rotifers/mL. These were subsequently transferred to 1.5-L Erlenmeyer flasks containing 1 L of saltwater adjusted to a salinity of 20 ppt.

Before larval feeding, the salinity was adjusted every hour to achieve a final salinity of 4 ppt, which is adequate for zebrafish larvae, as described [25]. To enrich the rotifers, microalgae at a cell concentration of 3.1 × 1010 cells/mL were added to the culture to reach a final concentration of 2.0 × 108 cells/mL and left under slight aeration for 14 h. On the following day, 2 h before larvae feeding, microalgae were added again to the culture for a final concentration of 2.0 × 108 cells/mL. Prior to feeding, a sample of 15 mL was collected from the enrichment flasks to determine the concentration of rotifers in the culture (Figure 1B), as described in Table 1.

### 2.3. Fish Housing

A broodstock of wild-type zebrafish AB (strain; ZFIN ID: ZDB-GENO-960809-7) was maintained at the Centre of Marine Sciences (CCMAR, Portugal), housed in 3.5-L polycarbonate tanks in a 980-L recirculation system (ZebTEC, Tecniplast, Italy). The fish room contained a controlled photoperiod with 14 h light/10 h dark cycle and an air temperature of 25.0 ± 1°C controlled by air conditioning. Water parameters were kept at 28.0 ± 0.5°C and pH 7.5 ± 0.1 with a conductivity of 750 ± 35 µS/cm. Water quality was maintained through a daily water change of 10% of total volume (98 L) and adjustment of conductivity and pH with artificial sea salts and sodium bicarbonate, respectively, using peristaltic pumps. The system is equipped with a complete filtration system: biological filtration (ceramic beads), mechanical filtration (pleated cartridge filters, 50 µm), carbon filtration (granular activated charcoal filter), and ultraviolet sterilization (180,000 µWs/cm^2^). The monitoring of nitrogen compounds was performed weekly and the values were kept below 0.1 mg/L for NO_2_^−^ and NH_4_^+^ and below 50 mg/L for NO_3_^−^. All the materials used in the filtration system were acquired from Tecniplast.

A group of males (*n* = 10) and females (*n* = 10), between 4 and 5 months were used to generate all the necessary larvae to perform the experiments. The spawning was performed with a sex ratio of 1:1, where males and females were placed in 1 L breeding tanks (Tecniplast, Italy) with a physical separator for 16 h before the reproduction event. In the first hour of light, the separator was removed and the fish were allowed to breed for 2 h. The eggs were counted and a total of 1800 eggs were collected and rinsed with clean system water. Eggs were incubated with E2 embryo medium containing 50 ppt of methylene blue (Sigma–Aldrich, Spain) at 28.0 ± 0.5°C in 1-L breeding tanks at a density of 200 eggs/L.

### 2.4. Fish Rearing and Diet

At 5 days postfertilization (dpf), larvae were randomly divided into triplicate groups (*n* = 100) in 1-L breeding tanks. Six experimental feeding regimes composed of rotifers enriched with three different species of microalgae, each one tested in two different biomass typologies (i.e., paste and freeze-dried; Table 1) were used. Each tank was provided with a daily total of 1500–1725 rots/mL, divided into two meals, with each meal containing 200–230 enriched rots/mL (Figure 1C). Additionally, one control group was fed with the microdiet standardized for zebrafish (Zebrafeed- ZF, Sparos Lda, Portugal). Fish tanks were cleaned daily by siphoning.

### 2.5. Fish Sampling

At 15 and 30 dpf, larvae from each tank were counted and the final survival rate was determined. A random sample of 15 larvae was collected from each replicate (*n* = 45), for determining standard length (SL) and dry weight (DW).

At 30 dpf, an additional sampling of 10 larvae per replicate (*n* = 30) was made for assessing mineral contents. For measuring SL, all individuals were photographed using a stereomicroscope (Leica MZ7.5, Leica, Germany) coupled with a color camera (DFC7000T; Leica, Japan). The collected images were further analyzed using Image J software [36]. These same larvae were then placed in a hoven at 60°C for 24 h to determine DW.

### 2.6. Chemical Analysis

#### 2.6.1. Proximal Composition for Microalgae and Enriched Rotifers

A sample of 1 mg of DW (microalgae or rotifers) was weighed on a microscale (Cubis, Sartorius, Germany) and placed in the Vario analyzer (Vario EL, Elemental Analyzer system, GmbH, Hanau, Germany) following the manufacturer's protocol. For each treatment, three samples of 1 mg were used. Thereafter, the percentage of protein was calculated from the multiplication of the nitrogen content percentage by 6.25, as described by Diniz et al. [37].

Total lipids were analyzed according to a modified protocol from Bligh and Dyer [38], described by Pereira et al. [39]. The microalgal pellet was extracted with a mixture of chloroform, methanol, and water (2:2:1) using an Ultra-Turrax (IKA-Werke GmbH, Germany) disperser for 2 min. Phase separation was achieved by centrifugation for 10 min at 3500 × *g*; the chloroform phase containing the lipids was collected and transferred to new vials (Normax, Portugal). A 0.7-mL sample of the lipid extract was then evaporated and the content of lipids was determined gravimetrically, using three replicates for each treatment.

Total ashes were determined by incineration of 50 mg of biomass of each sample using a Naberthern Controller B170 muffle furnace (Naberthern, Germany). Samples were incinerated for 8 h at 525°C, after which the total remaining weight was assessed and the amount of ash calculated according to Widbom [40]. The nitrogen free extract (NFE) content was determined by subtracting the weight of proteins, lipids, and ashes from the total DW of biomass.

#### 2.6.2. Fatty Acid Methyl Esters (FAMEs) Content

FAMEs were analyzed as previously described [35, 41] using a methodology adapted from Lepage and Roy [42]. Briefly, 10–20 mg of biomass were weighed and treated with 1.5 mL of derivatization solution (methanol/acetyl chloride, 20:1, v/v) in reaction vessels. A Bruker GC–MS (Bruker SCION 456/GC, Scion Instruments, UK) equipped with a ZB-5MS capillary column (30 m × 0.25 mm internal diameter, 0.25 μm film thickness, Phenomenex, USA) using helium as the carrier gas was used to analyze FAME at 1 mL min^−1^. The temperature program involved several steps, starting at 60°C for 1 min, followed by a ramp of 30°C for 1 min up to 120°C, then, a slower ramp of 4°C for 1 min up to 250°C, and finally a ramp of 20°C per minute up to 300°C, where it was held for 4 min. An injection temperature of 300°C was used. A Supelco37 Component FAME Mix (Sigma–Aldrich, Sintra, Portugal) was used as standard and separate calibration curves were generated for each of the FAME. Results are expressed as a percentage of the total fatty acid content.

### 2.7. Statistical Analysis

All data was tested for normality using Levene's test for the homogeneity of variances before being analyzed by one way ANOVA to determine differences between treatments, followed by Tukey's post hoc comparisons, with *p* ≤ 0.05. Results were expressed as mean ± standard deviation (SD). A principal component analysis (PCA) was used to analyze the biological parameters affecting the biological performance in each dietary treatment. IBM SPSS Statistics 25.0 (SPSS Inc., USA) software was used for data and statistical analysis, while GraphPad 9.4.0 (GraphPad Software, USA) was used for data visualization.

## 3. Results

### 3.1. Biochemical Composition of Microalgae and Rotifers

The proximal composition of microdiet, microalgae, and enriched rotifers is presented in Table 2. Amongst the microalgae studied, the total lipid content was highest in *Tisochrysis lutea* paste (TlPA) and freeze-dried *T. lutea* (TlFD), reaching 31.9% and 27.5% of DW, respectively. On the other hand, only 14.08% DW of lipids were registered in freeze-dried *Nannochloropsis oceanica* (NoFD). Regarding proteins, ZF had the highest content with 66.21% and the lowest amount was observed in *Tetraselmis chui* paste (TcPA) with 34.62%. Total carbohydrates (CHO) were highest in NFD (39.42% DW) and lowest in TlPA (3.59% DW; Table 2).

The higher content of total lipids was detected in rotifers enriched with *Nannochloropsis* paste (NoPA; 14.73% DW), while the lowest value (6.48% DW) was observed in TlFD. The rotifers enriched with freeze-dried *Tetraselmis chui* (TcFD) displayed the highest protein content with 40.28% DW, while only 21.94% DW of protein was obtained in rotifers enriched with NoFD. The ash content ranged from 54.52% DW in rotifers enriched using NoFD to 32.01% DW in rotifers enriched with TcFD. CHO were highest in the rotifers enriched with TlFD (19.78% DW) and lowest in those enriched with NoPA (14.82% DW).

Statistical analyses for the proximal parameters (%lipids, %protein, %ash, and %CHO) revealed significant differences among treatments, with effect sizes calculated using Cohen's d, demonstrating the magnitude of these differences.

Regarding lipids, the comparisons highlight substantial differences between treatments. For instance, TlPA and TcFD (*d* = 10.71) and TlFD and TcFD (*d* = 32.30) showed the largest effect sizes, indicating substantial differences in lipid composition. This underscores the significant influence of the form of microalga used for rotifer enrichment. Comparisons such as NoPA vs TlPA (*d* = −9.81) also highlight marked differences.

For protein content, the comparison between TlPA and TcPA (*d* = 7.84) revealed a very strong effect, showing important variations in the nutritional quality among treatments. Conversely, smaller differences, such as between NoPA and TcFD (*d* = −0.17), suggest similar protein compositions between these two treatments. Significant differences in the ash fraction were observed, particularly between NoFD and TcPA (*d* = −30.46) and NoPA and TcPA (*d* = −16.89). These values indicate substantial changes in the mineral fraction among samples, reflecting the specific contribution of each microalga to the final composition. Carbohydrates showed very large effects in comparisons such as NoFD and TlPA (*d* = 20.29) and NoFD and TlFD (*d* = 15.33), emphasizing notable differences in the energy content among treatments. On the other hand, comparisons such as TlFD and TcFD (*d* = −0.19) indicated minimal variation between these two treatments.

The fatty acid composition was quantified in rotifers enriched with microalgae (Table 3); it was observed that C22:6 (DHA) ranged from 6.18% in Zebrafeed to 0 in most the rotifer samples. The C20:4 (arachidonic acid (ARA)) ranged from 4.81% in rotifers enriched with TlPA, to 0.12% in the microdiet ZF. While C20:5 (EPA) ranged from 6.16% in rotifers enriched with NoPA to 1.90% in rotifers enriched with TlFD.

In addition, the content of omega 3 fatty acids ranged from 1.90% in rotifers enriched with TlFD to 9.33% in ZF, though omega 6 fatty acids ranged from 7.02% in rotifers enriched with TlPA to 32.66% in ZF. The smallest *n*−*3*/*n*−*6* ratio was observed in rotifers enriched with NoFD (0.18%) and highest in rotifers enriched with TlPA (1%).

#### 3.1.1. Larval Quality

**Figure 2:**
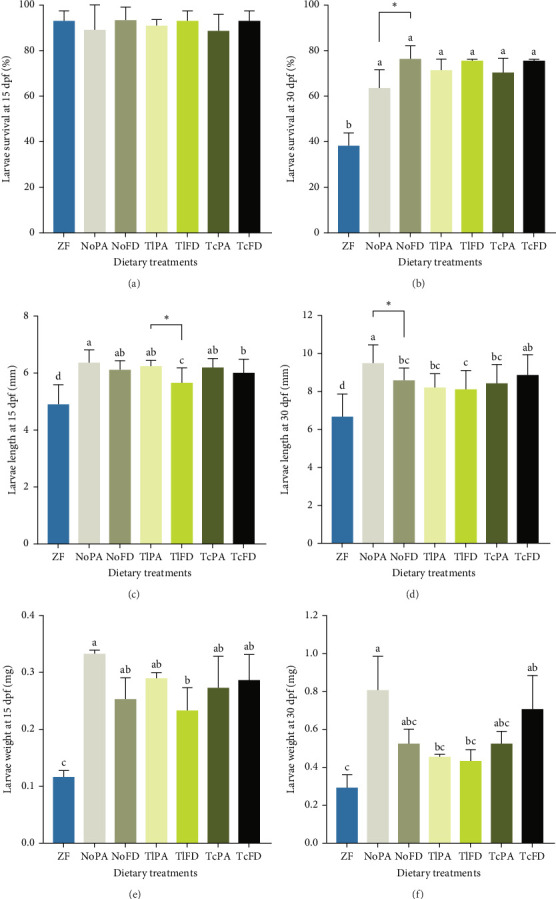
Zebrafish larvae survival was evaluated at 15 (A) and 30 days post-fertilization (dpf) (B). Total length at 15 dpf (C) and 30 dpf (D). Dry weight at 15 dpf (E) and 30 dpf (F). Data are expressed as mean ± SD. Letters indicate significant differences using one way ANOVA with Tukey's test (*p* ≤ 0.05 and 95% confidence interval; *n* = 15). Asterisks represent significant differences using a *t*-test (*p* ≤ 0.05). NoFD, *N. oceanica* freeze-dried; NoPA, *N. oceanica* paste; TcFD, *T. chui* freeze-dried; TcPA, *T. chui* paste; TlFD, *T. lutea* freeze-dried; TlPA, *T. lutea* paste; ZF, Zebrafeed.

Larval survival was quantified at 15 dpf (Figure 2A) and was observed that it ranged from 88.67% in larvae fed with TcPA to 93.33% in larvae fed with NoFD, without significant differences, while at 30 dpf (Figure 2B) larvae survival was significantly lower in larvae fed with ZF (38.33%) compared to the other groups. The survival on the other treatments ranged from 76.33% in larvae from the NoFD group to 63.67% in larvae fed with rotifers enriched with NoPA, without significant differences among the different treatments.

Growth was evaluated at 15 dpf and it was possible to observe that larvae fed with ZF had a lower length (4.91 mm), while larvae fed with NoPA had one of the highest larvae lengths (6.38 mm), even though not significantly different from larvae fed with NoFD (6.12 mm), TlPA (6.25 mm), and TcPA (6.21 mm; Figure 2C). Regarding the larvae length at 30 dpf, it was possible to observe that larvae fed with ZF had the smallest total length (6.69 mm) and NoPA had the highest length with 9.49 mm; however, this value was not significantly different from the larvae fed with NoFD (8.60 mm), TlPA (8.21 mm), and TcFD (8.89 mm), as shown on Figure 2D.

The DW of larvae fed with different dietary treatments was evaluated. It was possible to observe that larvae fed with ZF had the lowest weight (0.12 mg) and NoPA treatment promoted one of the highest weights with 0.33 mg at 15 dpf (Figure 2E). Similarly, at 30 dpf larvae from ZF treatment showed the lowest DW (0.29 mg), while larvae fed with NoPA had one of the highest weights (0.81 mg), even though only different from the weight of larvae fed with TlPA (0.46 mg) and TlFD (0.43 mg). Larvae fed rotifers enriched with TcFD weighed 0.71 mg and larvae fed rotifers enriched with NoFD and TcPA both weighed 0.53 mg (Figure 2F).

Regarding the differences amongst the different microalgae typologies, it was possible to observe that NoFD contributed to a higher larvae survival at 30 dpf when compared to NoPA. On the other hand, at the same time point (30 dpf) NoPA contributed to a higher larvae length than compared to larvae fed with NoFD. At 15 dpf, TlPA contributed to larvae with higher length than larvae fed with NoFD.

#### 3.1.2. PCA

PCA was performed for the treatments ZF, NoPA, NoFD, TlPA, TlFD, TcPA, and TcFD to assess the influence of the biological parameters measured, namely, survival, length, and weight at 15 and 30 dpf, using the data collected along the trial.

The PCA explains 63.44% and 20.60% of the data variability on the *x*- and *y*-axis, respectively. Larvae DW and length at 15 dpf were the major contributors to the *x*-axis, while the length at 30 dpf and survival at 30 dpf were the major contributors to the *y*-axis.

**Figure 3:**
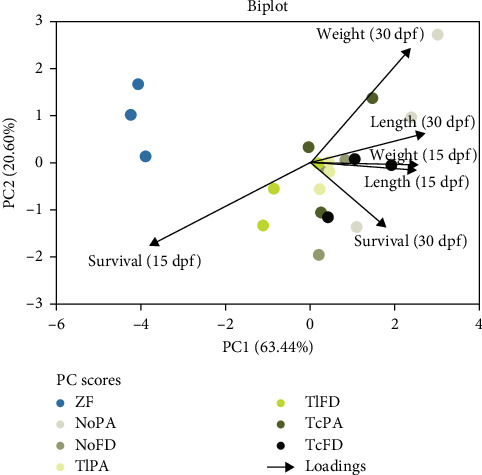
Principal component analysis (PCA) biplot grouped NoFD, *N. oceanica* freeze-dried; NoPA, *N. oceanica* paste; TcFD, *T. chui* freeze-dried; TcPA, *T. chui* paste; TlFD, *T. lutea* freeze-dried; TlPA, *T. lutea* paste; ZF, Zebrafeed. The plot is colored according to each treatment, for ZF blue, for NoPA gray, for NoFD dark gray, for TlPA yellow, for TlFD green, for TcPA dark green, and for TcFD black. Variables shown survival at 15 and 30 dpf, standard length at 15 and 30 dpf, and weight at 15 and 30 dpf.

It is interesting to observe that the weight and length at 15 dpf are positively correlated, based on their loads (Figure 3). The best principal component (PC) scores for weight and length at 30 dpf is for the NoPA treatment, while for the weight and length at 15 dpf the best PC corresponds to the TcFD treatment.

## 4. Discussion

Adequate nutrition and dietary protocols are essential for the enhancement of experimental reliability using any animal models. Thus, the understanding of zebrafish nutritional requirements and the standardization of its diet and feeding methodologies is of the utmost importance to increase the rigor and reproducibility among studies, consequently increasing its value as a translational model [8]. This study investigated the effect of rotifers enriched with different industrially produced microalgae species in two different typologies (e.g., freeze-dried and paste) on zebrafish larvae biological performance.

Lipids and proteins are the major constituents of fish and serve as a source of energy for growth, reproduction, and movement, being essential to achieve high growth and survival rates [43, 44]. In terms of proximal composition on rotifers, the highest percentage was found in rotifers enriched with NoPA with a value of 14.73% which lies in the range between 12% and 15% of total lipids, that have been identified in previous studies as optimal for zebrafish growth, all the other enrichments had lower values, which is not enough to contribute to a better growth [8, 45]. Regarding proteins, protein requirements are higher in the early life stages of teleosts, decreasing throughout development [46]. During the first days, including the beginning of the exogenous feeding, the larval digestive system is not entirely developed (altricial larvae), being unable to digest complex proteins [46]. A previous study pointed a narrow range between 37.0% and 38.2% of proteins in the diet to promote growth [47]. Among our treatments the closest value was 39.84%, found in rotifers enriched with TlPA. This may explain the good performance of larvae at 15 dpf fed with this treatment. However, the requirement in protein can be lower than previously reported, since the levels of protein from rotifers enriched with NoPA were only 34.38%, but this was the treatment that led to a higher growth in terms of length and weight at 15 and 30 dpf.

The dietary fatty acid composition also plays a very important role on growth performance. We observed that one of the highest contents of ARA (4.15%) was found in NoPA enriched rotifers. ARA content have been shown to have a positive correlation with larval survival and a significant improvement in the specific growth rates, body weight and total length [48]. EPA levels was also higher in this treatment and in most fish species lipids are favored as an energy source compared to proteins and carbohydrates. Additionally, this treatment is also characterized by high contents of long-chain PUFAs (LC-PUFAs) essential for cell membrane structure and function.

The developing fish embryos and larvae use for energy and metabolism the reserves of lipids in eggs [49]. According to the reported zebrafish larvae nutritional requirements, it is considered that zebrafish requires a 0.3–0.8 *n*−3/*n*−6 ratio to grow [29, 50], where in this study only two of our treatments met the proposed range, NoPA with 0.69 and TcFD with 0.4. We observed a significant difference between *Nannochloropsis oceanica* either in paste or freeze-dried, which may explain the different performances observed in larvae fed these two microalgae forms. Additionally, it is considered that zebrafish diets must contain a higher amount of *n*−6 than *n*−3 [51, 52] and this requirement was accomplished in all the treatments in our study.

Overall, it was possible to observe that the rotifers enriched with NoPA presented better performance when compared to the other treatments, considering the biological parameters studied (length, weight, and survival). We can conclude that *Nannochloropsis oceanica*, *Tisochrysis lutea*, and *Tetraselmis chui* have distinct and complementary biochemical compositions that support larval development and growth. *Nannochloropsis oceanica* is rich in EPA, an essential fatty acid critical for cell membrane integrity and larval growth [53]. *Tisochrysis lutea* provides high levels of DHA and carotenoids, which are crucial for enhancing larval survival, stress tolerance, and pigmentation, and *T. chui* is a notable source of essential amino acids and digestive enzymes, which aid in protein assimilation and support early developmental stages [54]. These microalgae not only contribute to the nutritional requirements of zebrafish larvae but also improve feed quality when used as enrichment in live feeds like rotifers, thereby enhancing growth performance and survival. The use of these microalgae, either individually or in combination, ensures a balanced dietary profile, meeting the specific nutritional demands of zebrafish at different life stages.

Altricial larvae exhibit an immature digestive system during their early life stages. In aquaculture, the early nutrition is primarily based on enriched live feeds, which are highly digestible and well-suited for the underdeveloped digestive systems of larvae, thereby enhancing their survival and growth compared to the use of microdiets during these stages [9]. Zebrafish begin feeding at 5 dpf, but their digestive system is still in its early stages. At this point, zebrafish lack acid digestion and most of their digestion occurs through peristaltic movements in the intestine and the action of intestinal lipases secreted by the exocrine pancreas [55, 56]. The use of live feeds, such as rotifers enriched with microalgae, in early life stages has been shown to improve survival in various fish species, including rainbow trout (*Oncorhynchus mykiss*), common carp (*Cyprinus carpio*), and zebrafish [57].

The use of life preys such as rotifers present several advantages, since they exhibit slow movement in the water column, making them easier for fish larvae to capture, have a high water content, providing a texture and consistency similar to the natural prey of fish larvae, and this similarity aids in the ingestion and digestion processes, promoting better feeding efficiency [58]. Moreover, when rotifers consume microalgae, they effectively pre-digest these organisms, which enhances the bioavailability of nutrients for fish larvae upon ingestion, facilitating better nutrient absorption [59, 60]. All these characteristics contribute to a higher growth performance when compared to microdiets.

In this study, zebrafish larvae fed with Zebrafeed exhibited significantly poorer growth in terms of length and weight at both 15 and 30 dpf, as well as lower survival rates at 30 dpf. Although zebrafish can be raised only on artificial diets [30, 61], previous research has consistently shown reduced growth when artificial diets are compared to live feeds, such as *Artemia* sp. [62]. For instance, Carvalho et al. [61] achieved 84% survival with artificial diets, which was comparable to the survival rate of larvae fed with *Artemia* nauplii, but the larvae were smaller in length. These findings align with the results of the present study, where the use of microdiets in early zebrafish larval stages resulted in the poorest growth and survival at the end of the experimental period compared to all other treatments. This outcome can be attributed to the poor digestibility of microdiets [29, 61].

For artificial diets to be effective, they must remain stable in water for an extended period to ensure that microparticles are available to the larvae. However, water-soluble vitamins degrade quickly in the water and leaching occurs due to the high surface-to-volume ratio of the particles [63]. Within a short time after being added to rearing tanks, artificial diets can lose up to 50%–95% of free amino acids and proteins, as well as up to 90% of water-soluble vitamins and minerals [63]. This degradation results in a nutritionally deficient food source, which not only hinders larval development but also leads to fouling in the tanks and reduced water quality [62].

*Nannochloropsis sp*. is the microalgal species most commonly used for enriching rotifers in aquaculture and is also a frequent choice in zebrafish production. As example, it was reported by Best et al. [25] that used this microalgae for rotifer enrichment and provided a density of 800 rot/mL during the first 4 days of zebrafish feeding under a polyculture system. With this protocol, they obtained larvae with 17.58 mm length and 93.60% survival at 30 dpf. A similar protocol was used by Lawrence et al. [17], but with different results since is this report, larvae reached only 9.49 mm of length and a survival of 98%. Both studies demonstrated higher rates of survival and growth when compared to our study. This can be the result of the different biochemical composition of the microalgae as well as the larval culture conditions used. In the previously mentioned studies, a larval density of 18 larvae/L was used, while in our study, larval culture started with 100 larvae/L and was gradually adjusted according to larvae growth. Fish density impacts growth, since high densities can lead to increased competition for resources such as food and oxygen, elevated stress levels, and impaired water quality, all of which negatively affect growth rates and overall health [64]. Conversely, low densities may improve growth performance by reducing competition and stress while ensuring better water quality [65]. In zebrafish, optimal stocking densities are crucial for maintaining growth and reproductive performance, as overcrowding has been associated with reduced growth rates and increased aggression [66].

It is worth mentioning that the biochemical composition of microalgae is not fully translated into the biochemical composition of rotifers, since these animals have shown to possess a dynamic metabolism with the consumption of important nutrients [67]. In the case of NoPA-enriched rotifers, it was possible to observe a similar content of lipids when compared to the microalgae. This fact was not observed in the other microalgae species studied, particularly in *T. lutea* showed 27.5%–31.0% of lipids, while the enriched rotifers with this microalga exhibited 6.48%–9.2% of lipids. This suggests that the lack of a cell wall in *T. lutea* promotes a high digestibility and metabolization of the lipids by the rotifers, as opposed to *N. oceanica* which presents a mechanically and enzymatically resistant cellulosic cell wall, most likely protected by an outer algaenan layer as observed for *Nannochloropsis gaditana* [68]. Therefore, this suggests that higher concentrations of *T. lutea* should be used to compensate the metabolism of rotifers and deliver the target nutritional profile for zebrafish larvae.

Microalgae proximal composition as well as the processing method (i.e., typology) may have an impact on larvae performance. Based on our results, and despite the absence of significant differences in the proximal compositions, it was possible to observe that the same species of microalgae using two distinct processing typologies (paste or freeze-dried), produced significant differences in the larvae performance. For example, TlPA contributed to higher growth at 15 dpf when compared to TlFD, while at 30 dpf NoPA also contributed to higher growth performance. Additionally, to this influence on larvae growth, other factors must be considered when choosing a microalgae product. Regarding the use of the product, microalgae that underwent a freeze-drying process did not solubilize as easily when added to the enrichment containers, as compared to the paste form. As a result: (i) it may have lowered the nutrient availability to the rotifers, (ii) it could have increased the debris content on the tanks, becoming more susceptible to faster lipid oxidation, and (iii) the higher debris content might have contributed to increased ammonia levels, affecting the rotifers population dynamics, which can ultimately result in the total loss of the batch culture. These three factors contributed to a better performance of paste over powder. Therefore, new methodologies must be developed to improve freeze-dried microalgae usage, for being more easily solubilized and digested by rotifers. In a small facility setting, the use of small amounts will increase the work and the margin of error. Consequently, pastes or liquid concentrates would be the most useful typologies to be used in rotifer nutrition and enrichment in the facilities dedicated to zebrafish research.

The production of microalgae pastes is more cost-effective for commercial producers, as it involves a simplified process. After scaling up, the biomass is harvested and centrifuged. In contrast, the production of freeze-dried biomass involves additional steps: after centrifugation, the biomass undergoes freeze-drying followed by grinding. These two extra steps increase commercial production costs by approximately 20% [69]. Furthermore, energy consumption rises during these processes. Specifically, freeze-drying requires the biomass to be placed in a freeze-dryer, where under vacuum, the water's vapor pressure is reduced, causing the water to transition directly from a solid (ice) to a gas (vapor) without passing through the liquid phase. This process demands careful temperature control, typically between −20 and −50°C, depending on the type of microalgae. Subsequently, the biomass is ground to achieve uniform texture and size, a process that is highly time-consuming. Despite these challenges, freeze-drying offers certain advantages, such as the ability to ship larger quantities of microalgae in the same space compared to microalgal paste and a significant extension of shelf-life. To achieve more sustainable and cost-effective production in the future, it is recommended that biomass should be dried using solar energy and the equipment used during cultivation, such as centrifuges, should be powered by renewable energy sources like wind or solar power. Moreover, the processes should be optimized to ensure a good and stable nutritional profile in order to be used as enrichment for live preys. These changes will decrease the production cost, and consequently, the microalgae cost, which will contribute to a higher incorporation of microalgae in feeds for zebrafish and aquaculture species. The increased incorporation of microalgae into diets will enhance growth, improve skeletal health, and boost reproductive performance in zebrafish [10, 30, 70].

Overall, this work allowed us to evaluate the biological performance of zebrafish fed with rotifers enriched with monocultures of microalgae. An improvement in all biological parameters in treatments using enriched rotifers was observed. Indeed, these results may be correlated with the fact that rotifer size and mobility are adequate to fish larvae size at mouth opening, while the microdiet has superficial tension and, consequently, decreased bioavailability to the larvae [9, 27]. Moreover, rotifers have digestive enzymes that partially digest microalgae and allow better assimilation of nutrients [71]. *Nannochloropsis oceanica* in paste was the form resulting in the best performance when used in enriched rotifer diets. It was also possible to observe that *Tetraselmis chui* led to a relatively good performant diet until 15 dpf. In the future, it would be interesting to test the use of blends composed of these two species of microalgae, maintaining *Nannochloropsis oceanica* as the main constituent of the blend since it appears to better meet the larval zebrafish nutritional requirements. Additionally, it would also be interesting to evaluate the impact of different microalgae species and blends on the growth, biochemical composition and skeletal malformations in the zebrafish larvae.

## 5. Conclusions

This study contributes to the optimization of the larval feeding methodology using rotifers, where rotifers enriched with microalgae have been shown to improve larvae growth both at 15 and 30 dpf and their survival at 30 dpf. Our data also demonstrated that the use of *Nannochloropsis oceanica* in paste, used for enrichment of rotifers, showed advantages on larvae growth over a microdiet regime and the other microalgae species tested. Overall, our results contribute to optimizing zebrafish larval rearing methodologies using rotifers to attain a higher growth performance and survival and generate knowledge for the determination of zebrafish nutritional requirements.

## Figures and Tables

**Table 1:** Microalgae species and biomass typologies used to enrich rotifers for feeding zebrafish larvae.

Microalgae	Treatment	Typology
*Nannochloropsis oceanica*	NoPA	Paste
*Nannochloropsis oceanica*	NoFD	Powder (freeze-dried)
*Tetraselmis chui*	TcPA	Paste
*Tetraselmis chui*	TcFD	Powder (freeze-dried)
*Tisochrysis lutea*	TlPA	Paste
*Tisochrysis lutea*	TlFD	Powder (freeze-dried)

*Note: Nannochloropsis oceanica* (No), *Tetraselmis chui* (Tc), and *Tisochrysis lutea* (TI) were used in paste (PA) and powder/freeze-dried (FD) form.

**Table 2:** Proximal composition of microdiet, microalgae, and enriched rotifers (*n* = 3).

Microdiet and microalgae					Enriched rotifers			
Treatment	Lipids (%)	Protein (%)	Ash (%)	CHO (%)	Lipids (%)	Protein (%)	Ash (%)	CHO (%)
ZF	15.41^c^	66.21^a^	13.00^c^	5.38^d^	—	—	—	—
NoPA	17.63 ± 1.47^c^	44.61 ± 4.72^b,d^	12.71 ± 0.97^c^	25.05 ± 7.16^b^	14.73 ± 3.02^a^	34.38^c^	36.07 ± 0.21^c^	14.82 ± 1.69^b^
NoFD	14.08 ± 5.52^c^	37.66 ± 3.77^c,d^	8.83 ± 0.52^d^	39.42 ± 2.28^a^	6.93 ± 1.09^c^	21.94^f^	54.52 ± 0.06^a^	16.61 ± 0.98^b,c^
TlPA	31.90 ± 1.44^a^	51.72 ± 0.40^b^	12.79 ± 0.83^c^	3.59 ± 1.02^d,e^	9.22 ± 3.03^b,c^	39.84^b^	33.00 ± 0.37^e^	17.92 ± 1.34^b^
TlFD	27.45 ± 0.28^a^	46.04 ± 1.12^b^	12.06 ± 1.07^c^	14.45 ± 0.33^c,d^	6.48 ± 0.76^c^	30.85^e^	42.88 ± 0.08^b^	19.78 ± 0.17^a,c^
TcPA	15.82 ± 1.71^c^	34.62 ± 3.06^c^	26.59 ± 0.64^a^	22.97 ± 4.14^b^	9.76 ± 0.17^a,c^	32.37^d^	35.08 ± 0.69^d^	16.04 ± 0.30^b^
TcFD	20.99 ± 0.04^b,c^	45.33 ± 3.61^b,d^	18.72 ± 0.24^b^	14.96 ± 3.80^c,d^	9.78 ± 1.38^a,c^	40.28^a^	32.01 ± 0.08^f^	17.93 ± 2.16^b^

*Note:* Data are expressed as mean ± letters indicate significant differences using one-way ANOVA followed by Tukey's test (*p*  ≤ 0.05 and 95% confidence interval). CHO, total carbohydrates.

Abbreviations: NoFD, *N. oceanica* freeze-dried; NoPA, *N. oceanica* paste; TcFD, *T. chui* freeze-dried; TcPA, *T. chui* paste; TlFD, *T. lutea* freeze-dried; TlPA, *T. lutea* paste; ZF, Zebrafeed.

**Table 3:** Fatty acid composition of microdiet (Zebrafeed) and rotifers enriched with different microalgae (*n* = 2).

FAME	Treatment						
	ZF	NoPA	NoFD	TlPA	TlFD	TcPA	TcFD
C16:0	28.12 ± 0.71	33.31 ± 1.04	35.81 ± 2.02	33.77 ± 0.19	37.11 ± 0.81	34.61 ± 1.66	32.46 ± 0.46
C18:0	7.67 ± 0.10	3.61 ± 0.05	6.21 ± 0.29	4.37 ± 0.13	4.71 ± 0.78	5.81 ± 0.42	4.46 ± 0.04
C16:1	3.06 ± 0.22	24.04 ± 0.55	18.78 ± 1.10	13.89 ± 0.02	20.10 ± 3.23	14.15 ± 0.12	13.50 ± 0.05
C18:1*c*	10.52 ± 0.12	9.26 ± 0.15	13.31 ± 0.05	16.65 ± 0.32	10.46 ± 0.40	17.29 ± 1.31	20.40 ± 0.13
C18:1*t*	2.29 ± 0.13	4.94 ± 0.01	6.10 ± 0.90	6.17 ± 0.00	6.03 ± 0.80	4.13 ± 0.30	5.05 ± 0.12
C20:1	1.03 ± 0.03	1.82 ± 0.18	2.27 ± 0.64	3.70 ± 0.15	1.77 ± 0.65	3.04 ± 0.22	3.27 ± 0.17
C22:1	2.54 ± 0.17	0.65 ± 0.10	0.57 ± 0.28	1.05 ± 0.00	—	0.80 ± 0.12	1.15 ± 0.02
C24:1	0.40 ± 0.03	0.23 ± 0.08	0.88 ± 0.49	0.63 ± 0.07	2.16 ± 0.07	1.06 ± 0.70	0.44 ± 0.43
C18:2*n*−6*c*	32.54 ± 0.12	5.81 ± 0.05	8.97 ± 1.93	0.51 ± 0.06	6.79 ± 0.73	10.64 ± 0.43	9.56 ± 0.08
C20:4*n*−6 (ARA)	0.12 ± 0.02	4.15 ± 0.09	0.85 ± 0.72	4.81 ± 0.00	2.23 ± 0.97	2.00 ± 0.01	2.57 ± 0.10
C20:5*n*−3 (EPA)	3.15 ± 0.08	6.16 ± 0.28	1.91 ± 1.16	6.09 ± 0.05	1.90 ± 1.33	2.87 ± 0.03	4.88 ± 0.19
C22:6*n*−3 (DHA)	6.18 ± 0.10	—	—	0.96 ± 0.27	—	—	—
∑*n*−3	9.33	6.59	1.91	7.04	1.90	2.87	4.88
∑*n*−6	32.66	9.96	9.82	7.08	9.01	13.95	12.15
∑*n*−3: ∑*n*−6	0.29	0.69	0.18	1.00	0.21	0.21	0.40
EPA: ARA	26.25	1.48	2.25	1.27	0.85	1.44	1.90

*Note:* Data are expressed as mean ± SD.

Abbreviations: NoFD, *N. oceanica* freeze-dried; NoPA, *N. oceanica* paste; TcFD, *T. chui* freeze-dried; TcPA, *T. chui* paste; TlFD, *T. lutea* freeze-dried; TlPA, *T. lutea* paste; ZF, Zebrafeed.

## Data Availability

The data that support the findings of this study will be made available from the corresponding author upon request.

## References

[1] Weisbroth S. H., Flatt R. E., Kraus A. L. (2020). *The Zebrafish in Biomedical Research: Biology, Husbandry, Diseases and Research Applications*.

[2] Kolb A., Hildebrandt F., Lawrence C. (2018). Effects of Diet and Social Housing on Reproductive Success in Adult Zebrafish, *Danio rerio*. *Zebrafish*.

[3] Lawrence C. (2007). The Husbandry of Zebrafish (*Danio rerio*): A Review. *Aquaculture*.

[4] Varga Z. M., Ekker S. C., Lawrence C. (2018). Workshop Report: Zebrafish and Other Fish Models—Description of Extrinsic Environmental Factors for Rigorous Experiments and Reproducible Results. *Zebrafish*.

[5] Williams M. B., Watts S. A. (2019). Current Basis and Future Directions of Zebrafish Nutrigenomics. *Genes & Nutrition*.

[6] Lawrence C., Best J., James A., Maloney K. (2012). The Effects of Feeding Frequency on Growth and Reproduction in Zebrafish (*Danio rerio*). *Aquaculture*.

[7] Powell M. L., Pegues M. A., Szalai A. J., Ghanta V. K., D’Abramo L. R., Watts S. A. (2015). Effects of the Dietary *ω*3: *ω*6 Fatty Acid Ratio on Body Fat and Inflammation in Zebrafish (*Danio rerio*). *Comparative Medicine*.

[8] Watts S. A., Powell M., D’Abramo L. R. (2012). Fundamental Approaches to the Study of Zebrafish Nutrition. *ILAR Journal*.

[9] Conceição L. E. C., Yúfera M., Makridis P., Morais S., Dinis M. T. (2010). Live Feeds for Early Stages of Fish Rearing. *Aquaculture Research*.

[10] Bravo-Tello K., Ehrenfeld N., Solís C. J. (2017). Effect of Microalgae on Intestinal Inflammation Triggered by Soybean Meal and Bacterial Infection in Zebrafish. *PLoS One*.

[11] Thépot V., Mangott A., Pirozzi I. (2016). Rotifers Enriched With a Mixed Algal Diet Promote Survival, Growth and Development of Barramundi Larvae, *Lates calcarifer* (Bloch). *Aquaculture Reports*.

[12] Makridis P., Olsen Y. (1999). Protein Depletion of the Rotifer *Brachionus plicatilis* During Starvation. *Aquaculture*.

[13] Naas K. E., N˦ss T., Harboe T. (1992). Enhanced 1st Feeding of Halibut Larvae (*Hippoglossus hippoglossus* L) in Green Water. *Aquaculture*.

[14] Raja R., Coelho A., Hemaiswarya S., Kumar P., Carvalho I. S., Alagarsamy A. (2018). Applications of Microalgal Paste and Powder as Food and Feed: An Update Using Text Mining Tool. *Beni-Suef University Journal of Basic and Applied Sciences*.

[15] Brown M. R. (2002). *Nutritional Value and Uses of Microalgae in Aquaculture*.

[16] Reed R. (2000). Microalgae Paste: A New Paradigm in Larval Feeds 76-77.

[17] Lawrence C., James A., Mobley S. (2015). Successful Replacement of *Artemia salina* Nauplii With Marine Rotifers (*Brachionus plicatilis*) in the Diet of Preadult Zebrafish (*Danio rerio*). *Zebrafish*.

[18] Martins S., Monteiro J. F., Vito M., Weintraub D., Almeida J., Certal A. C. (2016). Toward An Integrated Zebrafish Health Management Program Supporting Cancer and Neuroscience Research. *Zebrafish*.

[19] Lubzens E., Gibson O., Zmora O., Sukenik A. (1995). Potential Advantages of Frozen Algae (*Nannochloropsis* sp.) for Rotifer (*Brachionus plicatilis*) Culture. *Aquaculture*.

[20] Kwasek K., Wojno M., Iannini F., McCracken V. J., Molinari G. S., Terova G. (2020). Nutritional Programming Improves Dietary Plant Protein Utilization in Zebrafish *Danio rerio*. *PLoS One*.

[21] Cardoso C., Pereira H., Franca J. (2020). Lipid Composition and Some Bioactivities of 3 Newly Isolated Microalgae (*Tetraselmis* sp. IMP3, *Tetraselmis* sp. CTP4, and *Skeletonema* sp.). *Aquaculture International*.

[22] Yu C.-C., Chen H.-W., Chenb M.-J. (2010). Chemical Composition and Bioactivities of the Marine Alga *Isochrysis galbana* From Taiwan. *Natural Product Communications*.

[23] Gómez-Loredo A., Benavides J., Rito-Palomares M. (2016). Growth Kinetics and Fucoxanthin Production of *Phaeodactylum tricornutum* and *Isochrysis galbana* Cultures at Different Light and Agitation Conditions. *Journal of Applied Phycology*.

[24] Navarro N., Yúfera M. (1998). Population Dynamics of Rotifers (*Brachionus plicatilis* and *Brachionus rotundiformis*) in Semicontinuous Culture Fed Freeze-Dried Microalgae: Influence of Dilution Rate. *Aquaculture*.

[25] Best J., Adatto I., Cockington J., James A., Lawrence C. (2010). A Novel Method for Rearing First-Feeding Larval Zebrafish: Polyculture With Type L Saltwater Rotifers (*Brachionus plicatilis*). *Zebrafish*.

[26] Brown M. R., Jeffrey S. W., Volkman J. K., Dunstan G. A. (1997). Nutritional Properties of Microalgae for Mariculture. *Aquaculture*.

[27] Ferreira P. M. P. (2009). Manual De Cultivo e Bioencapsulação da Cadeia Alimentar Para a Larvicultura De Peixes Marinhos.

[28] Guinot D., Monroig Ó., Hontoria F., Amat F., Varó I., Navarro J. C. (2013). Enriched on-Grown *Artemia* Metanauplii Actively Metabolise Highly Unsaturated Fatty Acid-Rich Phospholipids. *Aquaculture*.

[29] Martins G., Diogo P., Santos T. (2020). Microdiet Formulation With Phospholipid Modulate Zebrafish Skeletal Development and Reproduction. *Zebrafish*.

[30] Martins G., Diogo P., Pinto W., Gavaia P. J. (2019). Early Transition to Microdiets Improves Growth, Reproductive Performance and Reduces Skeletal Anomalies in Zebrafish (*Danio rerio*). *Zebrafish*.

[31] Ferreira M., Cortina-Burgueño Á., Freire I., Otero A. (2018). Effect of Nutritional Status and Concentration of *Nannochloropsis gaditana* as Enrichment Diet for the Marine Rotifer *Brachionus* sp. *Aquaculture*.

[32] Koyande A. K., Chew K. W., Rambabu K., Tao Y., Chu D.-T., Show P.-L. (2019). Microalgae: A Potential Alternative to Health Supplementation for Humans. *Food Science and Human Wellness*.

[33] Watts S. A., D’Abramo L. R. (2021). Standardized Reference Diets for Zebrafish: Addressing Nutritional Control in Experimental Methodology. *Annual Review of Nutrition*.

[34] Bigagli E., D’ambrosio M., Cinci L. (2021). A Comparative in Vitro Evaluation of the Anti-Inflammatory Effects of a *Tisochrysis lutea* Extract and Fucoxanthin. *Marine Drugs*.

[35] Castaldi M. (2020). *Development of a Commercial Microalgae Enrichment of Live Feed for Zebrafish*.

[36] Schindelin J., Arganda-Carreras I., Frise E. (2012). Fiji: An Open-Source Platform for Biological-Image Analysis. *Nature Methods*.

[37] Diniz G. S., Barbarino E., Oiano-Neto J., Pacheco S., Lourenço S. O. (2011). Gross Chemical Profile and Calculation of Nitrogen-to-Protein Conversion Factors for Five Tropical Seaweeds. *American Journal of Plant Sciences*.

[38] Bligh E. G., Dyer W. J. (1959). A Rapid Method of Total Lipid Extraction and Purification. *Canadian Journal of Biochemistry and Physiology*.

[39] Pereira H., Páramo J., Silva J. (2018). Scale-up and Large-Scale Production of *Tetraselmis* sp. CTP4 (Chlorophyta) for CO2 Mitigation: From An Agar Plate to 100-m3 Industrial Photobioreactors. *Scientific Reports*.

[40] Widbom B. (1984). Determination of Average Individual Dry Weights and Ash-Free Dry Weights in Different Sieve Fractions of Marine Meiofauna. *Marine Biology*.

[41] Pereira H., Barreira L., Figueiredo F. (2012). Polyunsaturated Fatty Acids of Marine Macroalgae: Potential for Nutritional and Pharmaceutical Applications. *Marine Drugs*.

[42] Lepage G., Roy C. C. (1984). Improved Recovery of Fatty Acid Through Direct Transesterification without Prior Extraction or Purification. *Journal of Lipid Research*.

[43] Brett M. T., Müller-Navarra D. C. (1997). The Role of Highly Unsaturated Fatty Acids in Aquatic Foodweb Processes. *Freshwater Biology*.

[44] Tocher D. R. (2003). Metabolism and Functions of Lipids and Fatty Acids in Teleost Fish. *Reviews in Fisheries Science*.

[45] Sevgili H., Sezen S., Kanyilmaz M., Aktas Ö., Pak F. (2019). Dietary Protein Requirements of Zebrafish (*Danio rerio*). *Journal of Limnology and Freshwater Fisheries Research*.

[46] Aragão C., Conceição L. E. C., Martins D., Rønnestad I., Gomes E., Dinis M. T. (2004). A Balanced Dietary Amino Acid Profile Improves Amino Acid Retention in Post-Larval Senegalese Sole (*Solea senegalensis*). *Aquaculture*.

[47] Fernandes H., Peres H., Carvalho A. P. (2016). Dietary Protein Requirement During Juvenile Growth of Zebrafish (*Danio rerio*). *Zebrafish*.

[48] Atalah E., Hernández-Cruz C. M., Ganuza E. (2011). Importance of Dietary Arachidonic Acid for the Growth, Survival and Stress Resistance of Larval European Sea Bass (*Dicentrarchus labrax*) Fed High Dietary Docosahexaenoic and Eicosapentaenoic Acids. *Aquaculture Research*.

[49] Morais S., Mourente G., Ortega A., Tocher A. J., Tocher D. R. (2011). Expression of fatty acyl desaturase and elongase genes, and evolution of DHA: EPA ratio during development of unfed larvae of Atlantic bluefin tuna (Thunnus thynnus L.). *Aquaculture*.

[50] Meinelt T., Schulz C., Wirth M., Kürzinger H., Steinberg C. (2000). Correlation of diets high in n-6 polyunsaturated fatty acids with high growth rate in zebrafish (Danio rerio). *Comparative Medicine*.

[51] Cowey C. B., Sargent J. R. (1977). Lipid nutrition in fish, Comparative Biochemistry and Physiology Part B. *Comparative Biochemistry*.

[52] Watanabe T., Kiron V., Satoh S. (1997). Trace minerals in fish nutrition. *Aquaculture*.

[53] Adarme-Vega T. C., Lim D. K. Y., Timmins M., Vernen F., Li Y., Schenk P. M. (2023). Microbial Biofactories: A Promising Approach Towards Sustainable Omega-3 Fatty Acid Production. *Food Microbial Sustainability*.

[54] Borowitzka M. A., Beardall J., Raven J. A. (2016). *The Physiology of Microalgae*.

[55] Carten J., Farber S. (2009). A New Model System Swims Into Focus: Using the Zebrafish to Visualize Intestinal Lipid Metabolism in Vivo. *Future Lipidology*.

[56] Flores E. M., Nguyen A. T., Odem M. A., Eisenhoffer G. T., Krachler A. M. (2020). The Zebrafish as a Model for Gastrointestinal Tract-Microbe Interactions. *Cellular Microbiology*.

[57] Diogo P., Martins G., Eufrásio A., Silva T., Cabrita E. (2019). Selection Criteria of Zebrafish Male Donors for Sperm Cryopreservation. *Zebrafish*.

[58] Mona M. M., Asssem S. S., Srour M. T., Sharaf E. H., Mansour T. A. (2022). Compatible First Feeding for Larval Common Sole (*Solea solea*): Effect of Rotifer and Artemia Scheme Manipulation on Growth Performance and Survival. *Examines in Marine Biology & Oceanography*.

[59] Fu Z., Yang R., Zhou S., Ma Z., Zhang T. (2021). Effects of Rotifers Enriched With Different Enhancement Products on Larval Performance and Jaw Deformity of Golden Pompano Larvae *Trachinotus ovatus* (Linnaeus, 1758). *Frontiers in Marine Science*.

[60] Liu J., Xu W., Liu Y. (2022). Effects of Chitosan-Coated Microdiet on Dietary Physical Properties, Growth Performance, Digestive Enzyme Activities, Antioxidant Capacity, and Inflammation Response of Large Yellow Croaker (*Larimichthys crocea*) Larvae. *Fish Physiology and Biochemistry*.

[61] Carvalho A. P., Araújo L., Santos M. M. (2006). Rearing Zebrafish (*Danio rerio*) Larvae Without Live Food: Evaluation of a Commercial, A Practical and a Purified Starter Diet on Larval Performance. *Aquaculture Research*.

[62] Lawrence C., Harper C. (2011). The Laboratory Zebrafish.

[63] Rønnestad I., Yúfera M., Ueberschär B., Ribeiro L., Sæle Ø., Boglione C. (2013). Feeding behaviour and digestive physiology in larval fish: current knowledge, and gaps and bottlenecks in research. *Reviews in Aquaculture*.

[64] Andersson M., Kettunen P. (2021). Effects of Holding Density on the Welfare of Zebrafish: A Systematic Review. *Zebrafish*.

[65] Ellis T., North B., Scott A. P., Bromage N. R., Porter M., Gadd D. (2002). The Relationships Between Stocking Density and Welfare in Farmed Rainbow Trout. *Journal of Fish Biology*.

[66] Ribas L., Piferrer F. (2014). The Zebrafish (*Danio rerio*) as a Model Organism, With Emphasis on Applications for Finfish Aquaculture Research. *Reviews in Aquaculture*.

[67] Fernández-Reiriz M. J., Labarta U. (1996). Lipid Classes and Fatty Acid Composition of Rotifers (*Brachionus plicatilis*) Fed Two Algal Diets. *Hydrobiologia*.

[68] Scholz M. J., Weiss T. L., Jinkerson R. E. (2014). Ultrastructure and Composition of the *Nannochloropsis gaditana* Cell Wall. *Eukaryotic Cell*.

[69] Malcata F. X., Guedes A. C. (2018). *Marine Macro- and Microalgae: A Review*.

[70] Diogo P., Martins G., Gavaia P. (2015). Assessment of Nutritional Supplementation in Phospholipids on the Reproductive Performance of Zebrafish, *Danio rerio* (Hamilton, 1822). *Journal of Applied Ichthyology*.

[71] O’Brien-Macdonald K., Brown J. A., Parrish C. C. (2006). Growth, Behaviour, and Digestive Enzyme Activity in Larval Atlantic Cod (*Gadus morhua*) in Relation to Rotifer Lipid. *ICES Journal of Marine Science*.

